# Supplementary material for: Maternal health during the COVID-19 pandemic: Experiences of health workers in three Brazilian municipalities
Source: PLoS One. 2023 Aug 29;18(8):e0290068. doi: 10.1371/journal.pone.0290068 (PMC10464980; doi:10.1371/journal.pone.0290068)
Supplement: Attachment — Submitted filename: Response to reviewers.07.2023.docx [file pone.0290068.s002.docx]

**PLOS ONE**

**PONE-D-22-34357R1**

**Maternal health during the COVID-19 pandemic: experiences of health workers in three Brazilian municipalities**

**Dear Academic Editor and Reviewers,**

Thank you for the evaluation of the above-mentioned manuscript.

After careful reading, we considered suggestion made and responded the comment/suggestion in this document. We are resubmitting the manuscript to Plos One.

In addition to the ‘Rebuttal letter’, we have also included a ‘Revised Manuscript with Changes Highlighted’ and an unmarked version of the paper without tracked changes, according to the instructions received.

Thank you very much for the opportunity to review our work.

Kind regards,

The Authors

6. Review Comments to the Author

Reviewer #3: Thank you for your responses and modification of the manuscript.

In the findings section: Please integrate the quotes in the tables to the text on each theme as shown in the examples provided by reviewers in the previous round of comments. Integrating relevant quotes after each statement in the findings makes it possible for the reader to understand how those statements are supported by the primary data and how the conclusions related to the data were drawn.

We accepted the suggestion and modified the text to address thr questions raised by the reviewer. We appreciate the comments and inform you that the interviewees' speeches were inserted in the text for greater/better understanding of the context and our analyses.

We informed you that the phrase has been moved: The Niterói maternity hospital offered testing for COVID-19 (rapid test and PCR). (line 423-424)

In this sentence, we clarify that health professionals mentioned a behavior of pacientes and family members: In Niterói, difficulty in adherence to preventive measures by patients and their families was also mentioned. (line 404-405)

Correction: In the four maternity (line 557)
